# C+SCAV和SEAM两种预处理方案自体造血干细胞移植治疗非霍奇金淋巴瘤的疗效与安全性比较

**DOI:** 10.3760/cma.j.issn.0253-2727.2022.08.009

**Published:** 2022-08

**Authors:** 嘉琦 李, 莹 张, 洪智 耿, 思寻 贾, 小津 吴, 进 周, 香萍 宗, 贞 杨, 晓晨 陈, 超 马, 广华 陈, 海萍 戴, 彩霞 李, 德沛 吴

**Affiliations:** 苏州大学附属第一医院血液科，江苏省血液研究所，国家血液系统疾病临床医学研究中心，苏州大学造血干细胞移植研究所，苏州 215006 Jiangsu Institute of Hematology, The First Affiliated Hospital of Soochow University, National Clinical Research Center for Hematologic Diseases, Jiangsu Institute of Hematology, The First Affiliated Hospital of Soochow University, Institute of Blood and Marrow Transplantation, Soochow University, Suzhou 215006, China

**Keywords:** 克拉屈滨, 自体造血干细胞移植, 预处理, 非霍奇金淋巴瘤, Cladribine, Autologous stem cell transplantation, Conditioning regimen, Non-Hodgkin lymphoma

## Abstract

**目的:**

比较C+SCAV（克拉屈滨+司莫司汀+环磷酰胺+阿糖胞苷+依托泊苷）和SEAM（司莫司汀+依托泊苷+阿糖胞苷+美法仑）两种预处理方案在非霍奇金淋巴瘤（NHL）患者自体造血干细胞移植（auto-HSCT）中的疗效与安全性。

**方法:**

对2018年3月至2021年5月期间在苏州大学附属第一医院血液科接受auto-HSCT的61例NHL患者进行回顾性分析。

**结果:**

①61例NHL患者中，男37例，女24例；中位年龄48（21～66）岁，C+SCAV方案组19例，SEAM方案组42例，两组在基线特征方面差异无统计学意义（*P*>0.05）。②C+SCAV组、SEAM组中性粒细胞植入中位时间分别为10（8～15）d、9（7～16）d（*P*＝0.103），血小板植入的中位时间分别为13（9～22）d、12（7～30）d（*P*＝0.403），差异均无统计学意义。③C+SCAV组、SEAM组移植后1年无进展生存（PFS）率分别为（76.5±10.3）％、（78.4±6.8）％（*P*＝0.841），总生存（OS）率分别为100.0％、（95.2±3.3）％（*P*＝0.339）。④对于移植前达完全缓解（CR）状态的患者，C+SCAV组、SEAM组移植后1年PFS率分别为（92.3±7.4）％、（82.5±7.2）％（*P*＝0.406）。⑤C+SCAV组、SEAM组非血液系统严重不良反应（≥3级）发生率分别为10.5％（2/19）、40.5％（17/42）（*P*＝0.013），严重黏膜炎的发生率分别为5.3％（1/19）、31.0％（13/42）（*P*＝0.015），严重感染（≥3级）的发生率分别为10.5％（2/19）、19.0％（8/42）（*P*＝0.389）。

**结论:**

采用C+SCAV预处理方案auto-HSCT治疗NHL患者可获得与SEAM预处理方案相似的移植后1年后生存率，非血液系统严重不良反应发生率较低且不会增加严重感染的风险。

## Abstract (en)

**Objective:**

This study aimed to compare the efficacy and safety of cladribine, smustine, etoposide, cyclophosphamide, and cytarabine（C+SCAV）and smustine, etoposide, cytarabine, and melphalan（SEAM）conditioning regimens in autologous stem cell transplantation（auto-HSCT）for non-Hodgkin's lymphoma（NHL）.

**Methods:**

A retrospective analysis was conducted on 61 NHL patients who received auto-HSCT in the Department of Hematology, the First Affiliated Hospital of Suzhou University, from March 2018 to May 2021. The C + SCAV group and SEAM group had 19 and 42 patients, respectively.

**Results:**

① Among the 61 patients with NHL, 37 were male and 24 were female. The median age was 48（21–66）years old. There were 19 cases in the C+SCAV group and 42 cases in the SEAM group. There was no significant difference in the baseline characteristics between the two groups（*P*>0.05）. ② The median time to neutrophil and platelet engraftment in the C+SCAV cohort were 10（8–15）days and 13（9–22）days, respectively, which does not differ from the SEAM group（*P*＝0.103, *P*＝0.403）. ③ No differences existed between the two groups in terms of survival. The 1-year progression-free survival（PFS）was（76.5±10.3）％ for patients receiving C+SCAV and（78.4±6.8）％ for those who received SEAM（*P*＝0.841）. The 1-year overall survival was 100.0％ for the C+SCAV group and 95.2±3.3％ for the SEAM group（*P*＝0.339）. ④The 1-year PFS of patients with complete remission in the C+SCAV group was similar to those who in the SEAM group［（92.3±7.4）％ *vs*（82.5±7.2）％, *P*＝0.406］. ⑤ The incidence of non-hematological serious adverse events（≥grade 3）in the C+SCAV group and SEAM group were 10.5％（2/19）and 40.5％（17/42）（*P*＝0.013）, the incidence of severe mucositis was 5.3％（1/19）and 31.0％（13/42）（*P*＝0.015）, and the incidence of severe infection（≥ grade 3）was 10.5％（2/19）and 19.0％（8/42）（*P*＝0.389）, respectively.

**Conclusion:**

C + SCAV conditioning regimen appeared to be no different from the SEAM regimen in terms of survival. It can lower the incidence of SAE and does not increase the risk of severe infection. As a result, it can be used as an alternative conditioning regimen for lymphoma patients undergoing auto-HSCT.

自体造血干细胞移植（auto-HSCT）是高度侵袭性或复发/难治性（R/R）非霍奇金淋巴瘤（NHL）的标准治疗方法。其中，移植前预处理方案是auto-HSCT成功的关键因素之一。BEAM方案（卡莫司汀+依托泊苷+阿糖胞苷+美法仑）是目前应用最广泛的auto-HSCT预处理方案，约50％以上的患者可获得长期无进展生存[1]–[2]，但该方案不良反应较多，尤其是美法仑所致的黏膜炎使感染的风险增加，严重影响患者的生存质量及预后[3]，因而设计出一种高效低毒性的预处理方案是长期以来的客观需求。克拉屈滨是一种嘌呤核苷类似物，可以多途径杀伤肿瘤细胞，并与阿糖胞苷有协同增效作用[4]–[5]，在针对NHL患者的治疗中亦显示出良好的疗效与安全性[6]–[7]。因此，为进一步降低预处理的不良反应，本中心设计了C+SCAV预处理方案（克拉屈滨+司莫司汀+环磷酰胺+阿糖胞苷+依托泊苷）应用于auto-HSCT治疗NHL患者。当前卡莫司汀在国内持续供应短缺使得BEAM方案的应用难以实现，而司莫司汀同为亚硝基脲类药物，与卡莫司汀具有相似的抗肿瘤活性、临床毒性、骨髓抑制性及相似的代谢转归[8]，由司莫司汀+依托泊苷+阿糖胞苷+美法仑组成的SEAM预处理方案具有较好的疗效和安全性[9]–[11]。本研究对19例采用C+SCAV方案和42例采用SEAM预处理auto-HSCT的NHL患者进行回顾性分析，以期探讨C+SCAV预处理方案auto-HSCT治疗NHL的疗效和安全性。

## 病例与方法

一、病例资料

回顾性分析2018年3月30日至2021年5月30日我院61例采用C+SCAV或SEAM预处理方案auto-HSCT的NHL患者，其中，19例采用C+SCAV方案，42例采用SEAM方案。诊断分型采用2016年版WHO造血与淋巴系统肿瘤分类标准[12]，临床分期采用Ann Arbor分期标准，体能状态评分采用美国东部肿瘤协作组（ECOG）评分系统。预后风险分层采用国际预后指数，疗效评价采用2014年版Lugano非霍奇金淋巴瘤疗效评价标准[13]。

二、移植方案

预处理方案：①C+SCAV方案：司莫司汀250 mg/m^2^，−8 d；依托泊苷150 mg/m^2^每12 h 1次，−7 d～−5 d；环磷酰胺1.5 g·m^−2^·d^−1^，−4 d、−3 d；阿糖胞苷500 mg·m^−2^·d^−1^，−7 d～−3 d（克拉屈滨结束4 h后给药）；克拉屈滨6 mg·m^−2^·d^−1^，−7 d～−3 d。②SEAM方案：司莫司汀250 mg/m^2^，−8 d；依托泊苷200 mg·m^−2^·d^−1^，−7 d～−4 d；阿糖胞苷400 mg·m^−2^·d^−1^，−7 d～−4 d；美法仑140 mg/m^2^，−3 d。回输后第1 d开始予重组人粒细胞刺激因子（G-CSF）300 µg/d直到外周血中性粒细胞绝对计数（ANC）≥0.5×10^9^/L连续3 d。其他对症支持治疗主要包括成分输血、抗感染治疗、营养支持以及维持水、电解质、酸碱平衡等。

三、定义及标准

粒细胞植入定义为回输后ANC超过0.5×10^9^/L连续3 d。血小板植入定义为血小板计数超过20×10^9^/L连续3 d且脱离血小板输注。疗效评估采用2014年版Lugano非霍奇金淋巴瘤疗效评价标准，将疗效分为完全缓解（CR）、部分缓解（PR）、病情稳定（SD）以及疾病进展（PD）。无进展生存（PFS）期定义为自体造血干细胞回输至疾病复发、进展、末次随访或各种原因死亡的时间。总生存（OS）期定义为自体造血干细胞回输到死亡或末次随访的时间。从预处理的第1 d开始评估不良反应，评估标准依据CTCAE 5.0版。

四、随访

通过住院或门诊复查以及电话随访等形式进行随访，随访截至2021年11月30日。C+SCAV组的中位随访时间为17.5（6.2～39.9）个月，SEAM组的中位随访时间为22.7（0.5～43.5）个月。

五、统计学处理

采用SPSS 26.0和GraphPad Prism 8.0软件进行数据分析。分类变量以百分比表示，并使用卡方检验进行比较。服从正态分布的计量资料采用“mean±SD”表示，非正态分布的计量资料应用“中位数（范围）”表示，并分别采用*t*检验和Mann-Whitney *U*检验进行组间比较。采用Kaplan-Meier法进行生存分析。将单因素分析中*P*<0.2的变量纳入Cox回归进行多因素分析。*P*值小于0.05被认为具有统计学意义。

## 结果

一、患者基本临床特征

61例NHL患者中，男37例，女24例，中位年龄48（21～66）岁。其中C+SCAV组19例，SEAM组42例。78.7％的患者在移植前达到CR，16.4％的患者在移植前达到PR，而移植前疾病状态为SD的患者占4.9％。两组患者在年龄、性别、病理类型、Ann Arbor分期、ECOG评分、有无骨髓受累、LDH水平、IPI评分、既往治疗线数和移植前疾病状态等方面的差异均无统计学意义（表1）。

**表1:** C+SCAV和SEAM预处理方案用于非霍奇金淋巴瘤自体造血干细胞移植患者的临床特征

临床特征	C+SCAV组（19例）	SEAM组（42例）	统计量	*P*值
年龄［岁，*M*（范围）］	45（23~66）	48.5（21~66）	−0.156	0.876
性别［例（％）］			0.072	0.788
女	7（36.8）	17（40.5）		
男	12（63.2）	25（59.5）		
肿瘤细胞来源［例（％）］			1.545	0.214
T细胞淋巴瘤	8（42.1）	11（26.2）		
B细胞淋巴瘤	11（57.9）	31（73.8）		
病理类型［例（％）］			−0.479	0.632
DLBCL	9（47.4）	19（45.2）		
FL	2（10.5）	5（11.9）		
PTCL-NOS	6（31.6）	8（19.0）		
NKTCL	1（5.3）	3（7.1）		
MCL	0（0）	3（7.1）		
其他^a^	1（5.3）	4（9.5）		
Ann Arbor分期［例（％）］			1.120	0.290
Ⅰ+Ⅱ期	6（31.6）	8（19.0）		
Ⅲ+Ⅳ期	13（68.4）	34（81.0）		
有B症状［例（％）］	13（68.4）	19（45.2）	2.819	0.093
骨髓受累［例（％）］	6（31.6）	8（19.0）	1.120	0.290
LDH升高［例（％）］	8（42.1）	11（26.2）	1.545	0.214
ECOG评分［例（％）］			2.302	0.129
0~1分	15（78.9）	39（92.9）		
≥2分	4（21.1）	3（7.1）		
IPI评分［例（％）］			0.099	0.753
<3分	13（68.4）	27（64.3）		
≥3分	6（31.6）	15（35.7）		
既往治疗线数［例（％）］			0.057	0.811
≤2线	13（68.4）	30（71.4）		
>2线	6（31.6）	12（28.6）		
移植前疾病状态［例（％）］			−0.120	0.904
CR	15（78.9）	33（78.6）		
PR	2（10.5）	8（19.0）		
SD	2（10.5）	1（2.4）		
随访时间［月，*M*（范围）］	17.5（6.2~39.9）	22.7（0.5~43.5）	−1.503	0.133

注：C+SCAV：克拉屈滨+司莫司汀+环磷酰胺+阿糖胞苷+依托泊苷；SEAM：司莫司汀+依托泊苷+阿糖胞苷+美法仑；DLBCL：弥漫大B细胞淋巴瘤；FL：滤泡性淋巴瘤；PTCL-NOS：外周T细胞淋巴瘤非特指型；NKTCL：NK/T细胞淋巴瘤；MCL：套细胞淋巴瘤；CR：完全缓解；PR：部分缓解；SD：病情稳定。^a^包括高级别B细胞淋巴瘤2例、B细胞淋巴瘤2例、间变大细胞淋巴瘤1例

二、造血重建

C+SCAV组回输的中位单个核细胞（MNC）数为6.32（1.69～17.4）×10^8^/kg，中位CD34^+^细胞数为4.70（1.24～10.34）×10^6^/kg。SEAM组回输的中位MNC为6.24（1.27～20.15）×10^8^/kg，中位CD34^+^细胞为4.90（1.91～21.24）×10^6^/kg。两组回输MNC和CD34^+^细胞差异均无统计学意义（*P*＝0.918，*P*＝0.144）。

C+SCAV组所有患者均获得造血重建，而SEAM组有1例老年患者在造血重建前因肾功能衰竭死亡，其余患者均实现造血重建。C+SCAV组、SEAM组中性粒细胞植入中位时间分别为10（8～15）d、9（7～16）d（*P*＝0.103），血小板植入的中位时间分别为13（9～22）d、12（7～30）d（*P*＝0.403），差异均无统计学意义。

三、生存分析

截至2021年11月，C+SCAV组中6例出现疾病进展，未出现死亡；SEAM组中10例出现疾病进展，3例死亡。两组的中位PFS和OS期均未达到。C+SCAV组、SEAM组移植后1年PFS率分别为（76.5±10.3）％、（78.4±6.8）％（*P*＝0.841，图1A），OS率分别为100.0％、（95.2±3.3）％（*P*＝0.339，图1B），差异均无统计学意义。

**图1:**
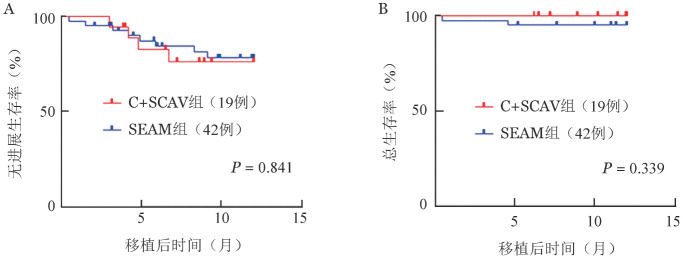
C+SCAV和SEAM预处理方案组移植后1年无进展生存（A）和总生存（B）曲线 C+SCAV：克拉屈滨+司莫司汀+环磷酰胺+阿糖胞苷+依托泊苷；SEAM：司莫司汀+依托泊苷+阿糖胞苷+美法仑

对移植前处于CR状态的患者进行分析，C+SCAV组和SEAM组的1年PFS率分别为（92.3±7.4）％和（82.5±7.2）％（*P*＝0.406，图2），无显著差异。而针对移植前未达到CR的患者，C+SCAV组4例中有3例出现疾病进展，SEAM组9例患者中有3例出现疾病进展。

**图2:**
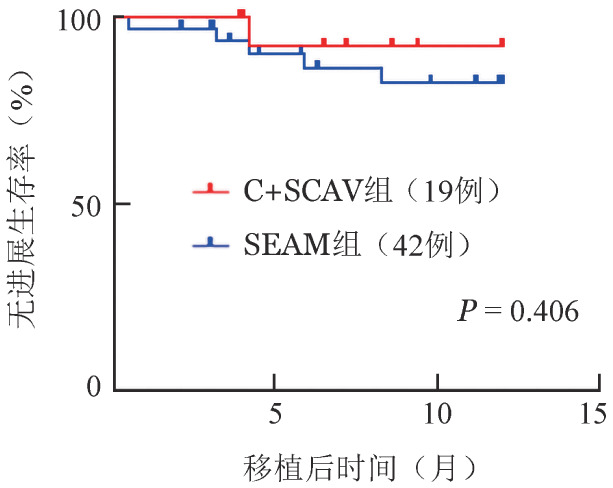
完全缓解后以C+SCAV或SEAM预处理方案行自体造血干细胞移植的非霍奇金淋巴瘤患者移植后无进展生存曲线 C+SCAV：克拉屈滨+司莫司汀+环磷酰胺+阿糖胞苷+依托泊苷；SEAM：司莫司汀+依托泊苷+阿糖胞苷+美法仑

四、安全性分析

对于血液系统不良反应，本研究中所有患者均发生Ⅳ级中性粒细胞减少和血小板减少；C+SCAV组、SEAM组分别有16例（84.2％）、31例（73.8％）患者发生3级及以上的严重贫血（*P*＝0.359）。

两种预处理方案相关的非血液系统不良反应主要包括黏膜炎、呕吐、腹泻、肝肾毒性、感染等。C+SCAV组有2例患者发生3级及以上的不良反应（严重不良反应），而SEAM组有17例患者发生严重不良反应，C+SCAV组的严重不良反应（≥3级）发生率低于SEAM组（10.5％对40.5％，*P*＝0.013）。黏膜炎是最常见的非血液系统不良反应，在80.3％（49/61）的患者中均有发生，C+SCAV组较SEAM组在黏膜炎的发生率上有着明显优势［63.2％（12/19）对88.1％（37/42），*P*＝0.028］。此外，黏膜炎也是最常见的严重不良反应，共有14例患者（23.0％）发生≥3级严重黏膜炎，在C+SCAV组、SEAM组中的发生率分别为5.3％（1/19）、31.0％（13/42）（*P*＝0.015）。对于化疗所致的恶心呕吐，C+SCAV组较SEAM组发生率也有明显减低［73.7％（14/19）对92.9％（39/42），*P*＝0.049］。此外，两组中分别有6例（31.6％）、15例（35.7％）患者出现肝肾毒性（*P*＝0.753），其中，1例患者在接受SEAM预处理方案后第14天死于化疗所致的肾功能衰竭，而在C+SCAV组中未见相关情况发生。C+SCAV组、SEAM组分别有2例（10.5％）、8例（19.0％）患者发生≥3级严重感染（*P*＝0.389）。

## 讨论

NHL是一类起源于淋巴系统恶性肿瘤，具有高度异质性[14]–[15]。对于高度侵袭性及R/R NHL，auto-HSCT是标准的挽救治疗手段，疗效优于传统化疗[16]–[17]。移植前预处理方案在auto-HSCT过程中发挥着重要的作用，理想的预处理方案不仅要有较好的疗效，而且相关不良反应少。克拉屈滨既可以作用于增殖期的细胞，又可以抑制静止期的细胞，通过多种途径促使肿瘤细胞凋亡[4],[18]。在一项联合克拉屈滨治疗套细胞淋巴瘤和惰性淋巴瘤的研究中，总缓解率达92％，且不良反应可控[19]。陈莉等[20]报道66例采用西达本胺+克拉屈滨+吉西他滨+白消安（ChiCGB）预处理方案进行auto-HSCT的NHL患者，移植后1年OS率、PFS率分别为84.85％、71.21％，初步揭示了克拉屈滨用于auto-HSCT预处理方案治疗NHL的可行性。此外，克拉屈滨能增加细胞内阿糖胞苷浓度，进一步增强两者的抗肿瘤效应[5],[21]。因此，为降低药物毒性、提高疗效，我们设计了C+SCAV预处理方案并与同期SEAM方案进行对照。

造血重建是auto-HSCT成功的关键，在一定范围内，造血重建时间越短患者生存预后越佳，移植所致死亡率明显下降。本项研究中，61例患者中有60例在移植后15 d内完成造血重建，粒系和巨核系重建的中位时间在两组之间无统计学差异。这一结果也与既往报道的大多数基于亚硝基脲的预处理方案[22]–[23]一致。表明C+SCAV方案能快速且有效实现造血重建。

2015年国际血液和骨髓移植研究中心（CIBMTR）针对3905例采用不同预处理方案进行auto-HSCT的NHL患者进行研究，移植后3年PFS率：BEAM组51％，CBV低剂量组52％，CBV高剂量组41％，BuCy组49％，含TBI方案组50％；移植后3年OS率：BEAM组64％，CBV低剂量组60％，CBV高剂量组52％，BuCy组59％，含TBI方案组59％；移植后1年治疗相关死亡率为4％～8％[1]。2018年EBMT淋巴瘤工作组纳入1149例NHL患者，针对BEAC和BEAM预处理方案进行匹配对照研究，BEAC组2年PFS率、OS率分别为63％、78％，BEAM组2年PFS率、OS率分别为63％、77％[24]。国内2019年发表的一项回顾性研究对比了改良BuCy和SEAM预处理方案在淋巴瘤auto-HSCT的疗效，结果显示两组患者的2年PFS率分别为79％、70％，2年OS率分别为81％、78％[8]。本研究中，C+SCAV组移植后1年PFS率、OS率分别为76.5％、100.0％，SEAM组分别为78.4％、95.2％，两组间在生存方面无明显统计学差异，也不低于既往报道，显示了C+SCAV方案的有效性及可行性。

黏膜炎是造血干细胞移植的一种严重且常见的不良反应，通常是由移植前大剂量化疗所致，相关研究表明在造血干细胞移植中黏膜炎的发生率约为80％，可影响造血重建、增加感染风险[25]–[26]。本研究结果显示，C+SCAV方案具有较低的黏膜炎发生率。

综上所述，本组病例结果显示，采用C+SCAV作为NHL患者auto-HSCT的预处理方案在生存方面与SEAM方案相似，并可降低严重非血液系统不良反应（尤其是黏膜炎）的发生率且未见重症感染风险的增加，提示C+SCAV方案可作为NHL患者auto-HSCT的备选预处理方案，本研究为回顾性，C+SCAV组例数有限，上述结论尚待验证。
